# Breastfeeding and female labor force participation: the probability of survival of children in Nepal under 3 years old

**DOI:** 10.1186/s13006-023-00560-6

**Published:** 2023-05-01

**Authors:** Kailash Timilsina, Yothin Sawangdee, Ravi Bhandari, Sirjana Tiwari, Ashmita Adhikari

**Affiliations:** 1 Institute for Population and Social Research, Mahidol University, Salaya, Thailand; 2 Gandaki University, Pokhara, Nepal; 3 School of Health and Allied Sciences, Pokhara University, Pokhara, Nepal; 4 School of Environmental Science and Sustainable Development, Kathmandu, Nepal

**Keywords:** Breastfeeding, Child survival, Female labor force participation

## Abstract

**Background:**

The number of breastfeeding mothers participating in a labor force to generate income has been increasing in Nepal. In this regard, the study aims to assess the survival of Under 3 children in Nepal from the mother based on their labor force participation and breastfeeding status.

**Methods:**

Data for the study were obtained from the Nepal Demographic and Health Survey 2016. The sample size of the study was 2,994 live births children, born in the last three years prior to the day of the interview. The robust hazard ratio and cox proportional hazard regression were conducted between dependent and independent variables with 95% confidence intervals (CIs) to conclude.

**Results:**

From a total of 2,994 live births, 85 children died within 36 months of birth. More than 80% of the non-working mothers were breastfeeding their children. The findings shows that the survival of children under-3 is positively associated with the interaction with the mother’s work and breastfeeding status (Hazard Ratio 0.428, 95% CI 0.24, 0.75), family structure (Hazard Ratio 1.511; 95% CI 1.37, 1.655), relationship with the household head (Hazard Ratio 0.452; 95% CI 0.311, 0.65), wealth quintiles (Hazard Ratio 0.390; 95% CI 0.33, 0.46), caste (Hazard Ratio 0.652; 95% CI 0.60, 0.69), and religion (Hazard Ratio 2.015; 95% CI 1.09, 3.70) with model CI 95%, Log pseudo likelihood = -521.39236, prob. χ2 = 0.005 and time at risk = 52,748.

**Conclusions:**

The highest rate of child survival was from the working mothers as well as continuing breastfeeding their children followed by mothers breastfeeding the child but not working, compared to mothers working but not breastfeeding the child, and mothers who were neither working nor breastfeeding their children respectively. This study provides clear evidence that breastfeeding is very important for the probability of survival of the child aged below 36 months and work of mother also have some positive impact on child survival. Employers should be encouraged to have a breastfeeding policy in the workplace through the establishment of a breastfeeding facility, and a flexible work schedule. At the same time government should also regulate the paid maternity leave and encourage societal support for the breastfeeding mothers.

## Background

Breastfeeding is considered as fundamentally important by the world’s health and scientific organizations for the infancy and child survival [1]. It was found that optimal breastfeeding could prevent around 12–13% deaths of children younger than five years, which is around 800 000 lives every year, in low- and middle-income countries [2, 3].

An increasing number of mothers from low- and middle-income countries are working outside the house to generate income and Nepal is not an exception [4]. The situation now is different from the past, as a mother’s primary role was mainly as a housewife, spending most of their time with children and household activities [5]. Working mothers are stopping breastfeeding and are feeding their children with additional milk and milk substitutes in most parts of Nepal [6]. But, working mothers can generate more income and can provide good nutrition and medical care to their children [4]. Therefore, this study’s aim is to find out how the interaction of breastfeeding and mother labor force participation effect the probability of survival among under-3 years old child in Nepal. Children under the age of three years are selected for the study, and according to “Nepal Demographic and Health Survey (NDHS) 2016”, breastfeeding has been documented for two-thirds of children under six months, and that children under three years breastfeed for an average of 30.5 months [7]. Mothers who work outside their houses and generate income are defined as working mothers. This study will find out the probability of the survival of children under-3 using the four different interactions between working mother and breastfeeding; (a) Mother currently not working and not breastfeeding her child; (b) Mother currently working and breastfeeding her child; (c) Mother currently working but not breastfeeding her child and (d) mother currently not working but breastfeeding her child.

## Methods

### Source of data

The study data were obtained from cross-sectional study of the Nepal Demographic and Health Survey 2016 (NDHS 2016). NDHS 2016 is a national representative survey implemented by New ERA under the aegis of the Ministry of Health (MOH) Nepal. The NDHS provides a comprehensive overview of population, maternal, and child health issues in Nepal. The structured questionnaire was used to collect those data after selecting the respondents from stratified random sampling methods, respondents were selected throughout Nepal in such a way that they were fully representative of the various areas of the country. All the children born in the last three years prior to the day of the interview and the characteristics of mothers who had given birth in the last three years were included in the study.

### Model specification

The dependent variables of the study were children under-3 mortality rate. The dependent variable was categorized as dummy variables 0 as the death of the child and 1 as the survival of the child. Under 3 mortality was defined as the death of a child from birth to 36 months of life. Children under-3 were included because most the mothers in Nepal breastfeed their child until the age of three. The total number of children under-3 in this study was 2,994. Independent variables of the study were the interaction between working mothers and breastfeeding. This interaction were classified in the 4 scenarios: (i) no work and no breastfeeding which is defined as the mothers who are currently not working and not breastfeeding their child; (ii) work and breastfeeding, which is defined as the number of working mothers who are breastfeeding their child; (iii) work but no breastfeeding, include the working mothers who did not breastfeed their child; (iv) no work breastfeeding, include the mothers who were not working but breastfeeding their child. Further, while defining breastfeeding status of a child who had died, two situations were taken into consideration; firstly, mothers were asked whether the child was breastfed at all? and secondly, whether the child was still breastfed at the time of death. Female Labor Force Participation was defined as mothers that were employed in income-generating work as follows; professional/technical/managerial, clerical sales and services, skilled manual, and unskilled manual.

Dependent variables of the study were chosen based on the fact that Nepal is a developing country with high child mortality. It ranks 67^th^ from below among 225 countries published by World Fact Book in 2017. Nepal also has very high child mortality if compared to its neighbouring countries such as Bangladesh, Maldives, Sri Lanka and a significantly higher mortality rate if compared to developed countries like the USA, Singapore, UK [8].

Independent variables of the study work of a mother are chosen based on the gender stratification perspectives that explain child mortality is mediated by woman statuses such as employed, woman health, education, and reproductive autonomy [9]. It was further supported by the theory of family development along with structure and feministic perspectives. According to this theoretical perspective, women experience changes over time such as when the family structure changes from an extended family to a nuclear family system. In a nuclear family, the decision-making power of a woman is high, independent of making decisions, and will involve in income-generating work [10].

The control variables of the study were maternal and child characteristics, education of the mother, family structure, age of the mother, the relationship of the mother with the household head, sex of a child, place of residence, types of cooking fuel along with wealth, caste, and the religion of the mother. The control variables of the study were chosen based on the well recognize flexible parametric framework developed based on hazard rate for analyzing infant and child mortality and Mosley and Chen framework of child mortality, these frameworks explain that individuals, households, and environmental characteristics have an impact on infant and child mortality at different ages [11, 12]. For caste of the mother, it is differentiated between Terai Caste’s mother and other caste mother as per NDHS. There is a severe caste-based discrimination in Terai regions of Nepal, bordering India. Among the 26 scheduled tribes and scheduled castes (Dalit) in Nepal, 19 live in Terai regions of Nepal [8, 13].

### Data analysis

The multivariate analysis for this study is exploring not only probability of dying via life table, but also examining the probability of dying using Cox-proportional hazard model. The interpretation of the result was based on hazard ratio (HR) value. The time variable for the analysis is the age of child when he/she had died or not died. Some socioeconomic variables may vary over time, and it is important to know the temporal effects of these variables on the failure time which is facilitated by Cox proportional hazard. Cox hazard was preferred because it takes into consideration both risk of death under-3 years of age of children when concentrating on time in each month [8]. The time variation here is measured by the current age of the child in months (months since birth for children who have died). Importantly, a robust cluster was also applied because one mother can have more than one child in this model. Robust cluster allows for correlation between observations within cluster therefore many children from same mother are correlated individually.

## Results and discussion

It can be seen in Table 1 that the total numbers of live birth of under-3 children were 2,994 in the last three years preceding the survey. Table 1 also shows the mother and child characteristics, which includes survival status of the child, sex of the child, number of births by mother, age of mother, education of mother, place of residence of mother, caste of the mother, wealth status of mother, relationship of the mother with household head, religion of mother, types of cooking fuel used in house and the mothers work and breastfeeding interaction. From a total of 2994 live births 85 children had died within 36 months of birth.

**Table 1:** Frequency distribution of maternal and child characteristics for under-3 death in Nepal 2012–2014

Mother and children characteristics	Under Three Children *N* = 2994	
	Frequency	Percentage
**Survival status of under 3 children**		
Alive Dead	2909 85	97.17 2.83
**Independent variables**		
**Maternal characteristics**		
**Number of births by mother**		
First birth More than 1 birth	1871 1123	62.55 37.45
**Age of mother at child death**		
Age 15–19 years Age 20 years and older	363 2631	12.10 87.90
**Education of mother**		
No education Have some education	883 2111	29.49 70.51
**Place of residence**		
Rural Urban	1298 1696	43.44 56.56
**Caste/Ethnicity**		
Terai caste Other castes	917 2077	30.61 69.39
**Wealth index**		
Other than rich Rich	2623 371	87.62 12.28
**Household relationship**		
Other than head of household Head of household	2517 477	84.12 15.88
**Work and breastfeeding interaction**		
No work no breastfeeding Work and breastfeeding Work but no breastfeeding No work breastfeeding	211 330 42 2411	7.00 11.08 1.40 80.52
**Family structure**		
Other than the nuclear family Nuclear family	2193 801	73.23 26.77
**Sex of child**		
Female Male	1389 1605	46.43 53.57
**Types of cooking fuel**		
Wood and other which generates smokes LPG and ignitable which do not generate much smoke	2386 608	79.71 20.29
**Religion**		
Other religion Hindu	409 2585	13.79 86.21

Looking at Table 2 it can be seen that the risk of death of under-3 children from a mother who does not have education is 28% higher as compared to mothers who have some education (HR 0.722, *P* value < 0.05, 95% CI 0.65, 0.79). This is supported by the findings from the survey data from 17 developing countries regarding the positive statistical association between maternal education and the health and survival of under-2 years children on post neonatal risk, undernutrition during the 3–23month period, and non-use of health services [14]. Maternal education has a strong positive impact on child survival. Uneducated mothers have the highest risk of child mortality. Mother’s education is a strong determinant of child survival in India, Tanzania, and Ethiopia [15–17].

**Table 2:** Results of the Cox hazard analysis for selected predictor variables associated with under-3 mortality, NDHS 2012–2014

Variables	Hazard Ratio	Robust Standard Error	Z	*P*-Value	95% Confidence Interval	
Education of mother	0.722	0.034	-6.79	0.000	0.657	0.793
Family structure	1.511	0.070	8.88	0.000	1.379	1.655
Birth by mother	3.835	0.048	106.84	0.000	3.742	3.931
Age of mother	1.525	1.279	0.50	0.614	0.294	7.895
Relationship with the household head	0.452	0.086	-4.15	0.000	0.311	0.658
Interaction work and breastfeeding	0.428	0.123	-2.95	0.003	0.243	0.752
Sex of child	0.859	0.450	-0.29	0.772	0.307	2.40
Place of residence	0.852	0.204	-0.66	0.507	0.532	1.365
Wealth	0.390	0.327	-11.22	0.000	0.331	0.460
Caste of mother	0.652	0.022	-12.15	0.000	0.608	0.698
Religion of mother	2.015	0.626	2.25	0.024	1.095	3.705
Cooking fuel	1.227	0.474	0.53	0.595	0.575	2.617

**Notes**: *n* = 2994, Log pseudo likelihood = -521.39236, prob. χ2 = 0.005, time at risk = 52748

Analysis time: current age of the child in months (months since birth for a dead child), SE: adjusted for two clusters in number of births by mother

Similarly, the risk of death of under-3 year old children other than nuclear family is 51% higher (HR 1.51, *p* value < 0.05, 95% CI 1.37, 1.655). The prevalence of malnutrition in the two rural areas of Peshawar is 35% in children under three years of age. Both socioeconomic factors (large family size of 87% respondents) and maternal factors were responsible for its high prevalence [18]. A study done in Pakistan found that the education of the mother, birth order number, preceding birth interval, size of child at birth, breastfeeding, and family size were found to have a significant effect on child mortality [19].

Further, the risk of death of under-3 children in the first baby is 84% higher as compared to the second and third baby of mothers. A Comparative Analysis of child mortality from 39 countries and a study from India found that mortality and risk of dying chance of first children compared to those born in middle and last-born child shown to be higher than average while other factors are controlled [20, 21]. Likewise, the risk of death of under-3 children from a mother who is not a household head is 55% higher (HR 0.45, *p* value < 0.05, 95% CI 0.31, 0.65). It was further supported by the theory of family development and structure and feministic perspectives, according to this theoretical perspective, women experience changes over time such as when the family structure changes from extended family to nuclear family system. In a nuclear family the decision-making power of a woman is high, independent of making decisions, and will involve income-generating work [10].

Further Table 2 also shows that the risk of death of under-3 children from a mother who does not work and does not breastfeed her child is 57% higher (HR 0.42, *p* value < 0.05, 95% CIs 0.24, 0.75). This was supported by the study done in 26 developing countries of sub-Saharan Africa, South Asia and the Middle East. Using demographic health survey findings from logistic regression analysis this study shows that maternal work is associated with a 24.5% higher risk of child mortality as compared to those mothers stay at home [22]. Furthermore, the risk of death of under-3 children from other than rich families is 61% higher (HR 0.39, *p* value < 0.05, 95% CI 0.331, 0.46). This result is justifiable as rich quintiles families can supply sufficient nutrients for the child and mother [23].

A study from North India found that socioeconomically advantaged children had significantly lower death rates [24] and women and children from the poor wealth quintile have a greater disadvantage in all indicators of women and child health [25]. Likewise, a State-Level Analysis of the correlation between wealth and health of India found a positive correlation between children’s health and the economic growth of the country from 1990 to 2007 [26].

The risk of death of under-3 children from the Terai caste of Nepal is 35% higher than other castes of Nepal. This result is strongly supported by the study done in north India, that found that the scheduled tribes and scheduled castes having poor wealth quintile and northern Indian women and children are at a greater disadvantage in all indicators of women and child health as compared to other groups [25]. Furthermore, the risk of death of under-3 children from the Hindu population is much lower than other religions, as Hindu people enjoy more social support and access to formal health facilities. The study in Mozambique also found that in the predominantly Christian area, church membership increases the likelihood of getting social support and high access to healthcare facilities, [27]. Likewise, findings from Nigeria suggest that age, place of residence, educational status, wealth index, and religion of fathers and mothers are major determinants of childhood mortality [28].

**Fig. 1:**
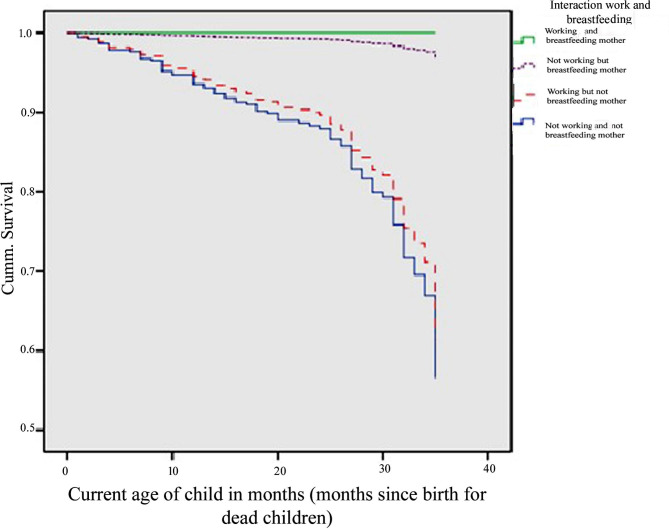
Prediction of survival of child by the caste of mother and paid maternity, 2012–2014, Nepal

Figure 1 presents the Cox proportional hazards regression analysis which presents the probability of survival of children below 3 years of age based on the status of the interaction of the mother’s work and her breastfeeding interactions. Interactions of the mother’s work and breastfeeding status are divided into 4 categories and the probability of survival of the child is clearly shown in the Figure. It can be seen from Fig. 1 that the survival of children from mothers who were working and breastfeeding was higher than other categories. The Figure also shows that the survival status of the children from the mothers who were not working and who were not breastfeeding their children was the lowest among the 4 categories, as shown in the blue line. Further child survival status of the mothers who were breastfeeding but not working is second after mothers who were both breastfeeding and working.

Furthermore, the survival status of the children of mothers who were working but not breastfeeding was below mothers who were both working and also breastfeeding their babies. The survival status was above the mother who was working but not breastfeeding their child and the mother who was neither working nor breastfeeding.

### Limitation of the study

Data used here may have some recall bias. For example, dependent variables may have recall bias as respondents are asked about the events as far back as three years before the day question is asked. Another issue regarding dependent variable is that survey is cross-sectional, so it does not follow the children until they reach three years old, and unfortunately, some children are likely to die before reaching three years of age although they are alive during the survey time and date. It is also possible that whatever led to the child’s death also led to cessation of breastfeeding; thus, cause and effect could be reversed.

## Conclusions

The risk of death of an under-3 child is significantly associated with the family structure, birth order by the mother, relationship with the household head, interaction with work and breastfeeding, wealth quintiles, caste and religion of the mother.

The study provides clear evidence that breastfeeding is very important for the probability of survival of the child aged below 36 months in Nepal, and work of mother also has some positive impact on child survival. Therefore, all mothers should be encouraged to participate in both working and to continue to breastfeed their children. Further, Nepal should develop a workplace policy in such a way that working women in both formal and informal sectors can continue breastfeeding their children. Such policies include declared breaks for breastfeeding in workplaces, onsite childcare, safe store breast milk, safe physical layout with breastfeeding facilitators, workplace breastfeeding advocacy, and affordable childcare near her workplaces. Employers should be encouraged to have a breastfeeding policy in the workplace through the establishment of a breastfeeding facility, and a flexible work schedule. At the same time government should also regulate the paid maternity leave and encourage societal support for the breastfeeding mothers.

## Data Availability

The study data were obtained from the Nepal demographic health survey (NDHS) 2016 which was done by Ministry of Health, Nepal hence ethical approval is not required for the analysis of data from Nepal demographic health survey (NDHS) 2016.

## References

[1] WHO, Breastfeeding. 2018. https://www.who.int/health-topics/breastfeeding#tab=tab_1. Accessed 10 Feb 2021.

[2] Bhutta ZA, Labbok M (2011). Scaling up breastfeeding in developing countries. The Lancet.

[3] Black RE, Victora CG, Walker SP, Bhutta ZA, Christian P, De Onis M, Ezzati M, Grantham-McGregor S, Katz J, Martorell R, Uauy R (2013). Maternal and child undernutrition and overweight in low-income and middle-income countries. The Lancet.

[4] Brauner-Otto S, Baird S, Ghimire D (2019). Maternal employment and child health in Nepal: the importance of job type and timing across the child’s first five years. Social Science & Medicine.

[5] Khanal U (2019). Role of women for making household decision in nepalese societies. ACADEMICIA: An International Multidisciplinary Research Journal.

[6] Sharma I, Khadka A (2019). Assessing the level of knowledge and practice of breastfeeding among factory working mothers in Kathmandu, Nepal. J Health Res.

[7] New ERA, Ministry of Health, Nepal (2017). 2016 Nepal demographic and Health Survey Key Findings.

[8] Timilsina K, Sawangdee Y, Hunchangsith P, Rittirong J (2019). Female labor force participation, paid maternity, caste system and under-5 mortality in Nepal. J Health Res.

[9] Shen C, Williamson JB (1997). Child mortality, women’s status, economic dependency, and state strength: a cross-national study of less developed countries. Soc Forces.

[10] Carter BE, McGoldrick ME. The changing family life cycle: a framework for family therapy. Gardner Press; 1988.

[11] Mutunga CJ. Environmental determinants of child mortality in Kenya.Health Inequality and Development. 2011:89–110.

[12] Van der Klaauw B, Wang L. Child mortality in rural India. *World Bank Publications* 2004. Available at SSRN 610326.

[13] Central Bureau of Statistics, Nepal. *National population and housing census 2011* National Report.2011.

[14] Bicego GT, Boerma JT (1993). Maternal education and child survival: a comparative study of survey data from 17 countries. Soc Sci Med.

[15] Armstrong Schellenberg JR, Nathan R, Abdulla S, Mukasa O, Marchant TJ, Tanner M (2002). Risk factors for child mortality in rural Tanzania. Tropical Med Int Health.

[16] Das Gupta M (1990). Death clustering, mothers’ education and the determinants of child mortality in rural Punjab, India. Popul Stud.

[17] Fenta SM, Fenta HM (2020). Risk factors of child mortality in Ethiopia: application of multilevel two-part model. PLoS ONE.

[18] Gul R, Kibria Z (2013). Prevalence and predeterminants of malnutrition in children under 3 years of age in the two rural communities of Peshawar. Khyber Med Univ J.

[19] Ahmed Z, Kamal A, Kamal A (2016). Statistical analysis of factors affecting child mortality in Pakistan. J Coll Physicians Surg Pakistan.

[20] Hobcraft JN, McDonald JW, Rutstein SO (1985). Demographic determinants of infant and early child mortality: a comparative analysis. Popul Stud.

[21] Mishra SK, Ram B, Singh A, Yadav A (2018). Birth order, stage of infancy and infant mortality in India. J Biosoc Sci.

[22] Amir-ud-Din R, Zafar S, Muzammil M, Shabbir R, Malik S, Usman M (2021). Exploring the relationship between maternal occupation and under-five mortality: empirical evidence from 26 developing countries. Eur J Dev Res.

[23] Sahu D, Nair S, Singh L, Gulati BK, Pandey A, Levels (2015). Trends & predictors of infant & child mortality among scheduled tribes in rural India. Indian J Med Res.

[24] Krishnan A, Dwivedi P, Gupta V, Byass P, Pandav CS, Ng N (2013). Socioeconomic development and girl child survival in rural North India: solution or problem?. J Epidemiol Community Health.

[25] Jungari S, Chauhan BG (2017). Caste, wealth and regional inequalities in health status of women and children in India. Contemp Voice Dalit.

[26] Coffey D, Chattopadhyay A, Gupt R. Wealth and health of children in India: a state-level analysis.Economic and Political Weekly. 2014;64–70.

[27] Cau BM, Sevoyan A, Agadjanian V (2013). Religious affiliation and under-five mortality in Mozambique. J Biosoc Sci.

[28] Yaya S, Ekholuenetale M, Tudeme G, Vaibhav S, Bishwajit G, Kadio B (2017). Prevalence and determinants of childhood mortality in Nigeria. BMC Public Health.

